# Achieving the sustainable development goals: a case study of the complexity of water quality health risks in Malawi

**DOI:** 10.1186/s41043-016-0057-x

**Published:** 2016-07-15

**Authors:** Rochelle Holm, Philip Wandschneider, Allan Felsot, Golden Msilimba

**Affiliations:** 1 School of Earth and Environmental Sciences, Washington State University, 2710 Crimson Way, Richland, WA 99354 USA; 2 Mzuzu University, P/Bag 201, Mzuzu 2, Malawi; 3 School of Economic Sciences, Washington State University, Hulbert 103h, Pullman, WA 99164 USA; 4 Department of Entomology, Food & Environmental Quality Lab, Washington State University, 2710 Crimson Way, Richland, WA 99354 USA

**Keywords:** Communication, Groundwater, Human dimensions, Malawi, Risk, Water quality

## Abstract

**Background:**

Suppose 35 % of the households with children under 5 years of age in a low-income suburban neighborhood in a developing country have diarrhea where improved water sources are available. Clearly, something is amiss—but what? In addition to focusing on the need to examine water quality among water sources that meet the ‘improved’ category when assessing health risk, the relative importance of the range of transmission routes for diarrhea is unknown. In Malawi, relevant baseline data affecting human health are simply not available, and acquiring data is hampered by a lack of local analytical capacity for characterizing drinking water quality. The objective of this work is to develop a risk communication program with partnership among established regional development professionals for effectively meeting the sustainable development goals.

**Methods:**

A field study was conducted in the city of Mzuzu, Malawi, to study water quality (total coliform and *Escherichia coli*) and human dimensions leading to development of a public health risk communication strategy in a peri-urban area. A structured household questionnaire was administered to adult residents of 51 households, encompassing 284 individuals, who were using the 30 monitored shallow wells.

**Results:**

The water quality data and human dimension questionnaire results were used to develop a household risk presentation. Sixty-seven percent and 50 % of well water and household drinking water samples, respectively, exceeded the WHO health guideline of zero detections of *E. coli*. Technology transfer was advanced by providing knowledge through household risk debriefing/education, establishing a water quality laboratory at the local university, and providing training to local technicians.

**Conclusions:**

Communicating the science of water quality and health risks in developing countries requires sample collection and analysis by knowledgeable personnel trained in the sciences, compiling baseline data, and, ultimately, an effective risk presentation back to households to motivate behavioral changes to effectively protect future water resources and human health.

## Background

Suppose 35 % of the households with children under 5 years of age in a low-income suburban neighborhood in a developing country have diarrhea. A further look shows neighborhood water sources meeting United Nation’s Millennium Development Goals are available [1]. The technical goal was satisfied, but the results are not satisfactory when considering the sustainable development goals (SDGs) [2]. Clearly, something is amiss—but what? More information is needed, but in Malawi, relevant baseline environmental data affecting human health are simply not available, and acquiring data from the drinking water ladder in response to the new SDGs is hampered by a lack of local analytical capacity for characterizing drinking water quality. We present methods to develop information needed in this and similar cases and results specific to Malawi. In our approach, applied research on social practices and physical circumstances is coordinated and simultaneous.

The World Health Organization (WHO) finds over 3000 children under 5 die each year in Malawi of diarrheal diseases [3], which are often associated with unsafe drinking water. In addition to focusing on the need to examine water quality among water sources that meet the ‘improved’ category when assessing health risk, the relative importance of the range of transmission routes for diarrhea is unknown. Handwashing has been shown to be one of the simplest methods to prevent spreading bacteria responsible for most waterborne diarrheal diseases [4, 5]. We propose to use a case study in Malawi to illustrate some of the obstacles to attaining safe water and to suggest strategies to facilitate attaining the SDGs. We will show the critical, but lofty, goal of safe water can be only partly achieved by improvements in water supply technology itself. Recent evidence has shown the water quality impact of providing information to communities is equivocal [6].

Before we return to the puzzle described in the opening paragraph, we cite a case at the foundation of epidemiology for precedent and inspiration. In the 1850s, the germ theory and miasma theory of disease were still rivals. Many scientists and officials in Europe believed cholera was an airborne illness following the miasma theory of disease [7]. Whereas advances in microscopy led to evidence supporting the germ theory for some diseases, the organism causing cholera was too small to detect easily at the contemporary level of technology. Johnson tells the sad and fascinating story of how officials laboring under the miasma theory actually worsened conditions with respect to cholera by creating sewer lines that reduced the “London stench” (miasma) yet inadvertently poisoned water sources. Following the 1854 epidemic in the Soho district of London, the coordinated efforts of Dr. John Snow’s careful cartographical analysis and local clergyman Henry Whitehead’s thorough in-person case interviews joined to locate the source of contamination in water, in fact, in one specific standpipe. By careful observation and documentation of the occurrence of cholera and use of public wells, Snow linked oral ingestion, rather than air transmission, to cholera. Eventually, restricting access to wells linked to cholera hot spots halted the epidemic [7]. Open inquiry and coordinated technical and behavioral research combined to identify and, ultimately, stop the outbreak—and eventually led to the discovery of the cause of cholera. In Malawi, even in the few cases where information about poor water quality is available in published reports or scientific journals, it is rarely communicated in a useful way to local households. Also, Mulwafu and Msosa [8] argue access to a supply of uncontaminated water in Malawi has deep socioeconomic ramifications.

The remainder of this paper presents a case study of an integrated approach to water quality risk and disease prevention towards meeting the ultimate objectives (as well as the technical measures) of the SDGs in Malawi [9]. For convenience, we label the strategy the Risk-Behavior-Communication & Technology Transfer or RBCTT program. The RBCTT program includes four components: (1) technical risk assessment, (2) local population behavioral human factor survey, (3) two-way communication, and (4) technology transfer.

### Water quality, disease, and risk communication

No perfect recipe exists for a risk communication program, partly because the perception of risk is a complex, multidimensional, and individual matter [10]. Risk communication programs may be implemented by governmental, nongovernmental, or grass roots organizations. A number of key criteria for sound programs have been identified including that information must be simple and easily communicated as well as continually updated and refined [11]. For natural disasters, Weinstein [12] points out three major elements in understanding and promoting risk reduction behavior: Perceptions of risk, characteristics of people, and incentives. These elements can be applied to protecting drinking water. For example, an individual who experiences waterborne diarrhea may be prompted to adopt protective measures.

The perception of risk and the propensity to adopt protective measures are often clouded by a number of human perceptual and judgment biases. These biases can lead to subjective and sometimes inaccurate  risk perceptions. Factors which influence risk perceptions, memory of risk events, and subsequent actions include the following [13]:

- *Salient and recent* events are more memorable than earlier and typical events
- Nature of *media* coverage of events (style and frequency as well as accuracy)
- *Worst-case scenarios* have more influence than typical events
- Circumstances or *framing* of risks changes perceptions
- Risk *perceptions change slowly*, especially when they conflict with other beliefs

Risks are statistical and humans have trouble understanding statistical concepts [13]. For example, individuals tend to think in terms of events and not colony forming units per 100 ml sample as used for *Escherichia coli* reporting. Hence, factors like those listed above can reinforce outdated information or influence people to adopt inaccurate and dangerous perceptions, attitudes, and practices. Finally, *cultural beliefs* are an important factor in water management, especially concerning the role of women, local history, and local politics [14, 15]. Analysts must attend to the actual concerns, attitudes, and opinions of the target audience in order to design effective public health risk communication programs [16]. Qualities of body language, openness, and emotional tone are also essential for gaining trust in an audience. One study in England showed the two most trusted sources of risk information were friends (80 %) and family (90 %), which illustrates the importance of people in risk management [16].

## Methods

Malawi has an estimated population of 17.3 million, of which 90 % of the population uses an improved drinking water source, but only 41 % of the population has access to an improved sanitation source [17]. The Malawi National Water Policy states the government vision is “Water and Sanitation for all, always.” The water policy also states the need for capacity building at Malawian universities and ambitiously declares a policy to “establish an accredited water and sanitation institution” [18].

This study was undertaken in the city of Mzuzu, Malawi (11.408° S 34.001° E) from January to March 2012. Mzuzu is the major urban center in northern Malawi with the population reported at 133,968 [19]. Mzuzu University is a public institution and admitted its first students in January 1999. There is municipal piped water supply service in the peri-urban areas of Mzuzu, Malawi, but households still use many traditional, shallow dug wells.

The study neighborhood, area 1B, is characterized as a high density and unplanned residential settlement at the edge of the city boundary. While the land is owned by the Mzuzu City Council, official boundaries do not exist. Establishment of the area occurred sometime before 1995 (anonymous civil servant, Malawi Government Department of Surveys, personal communication). Before 1998, groundwater was the only source of water in area 1B. The first municipal piped water supply to area 1B was established in 1998, but was discontinued in 2000 due to high demand in another nearby area. From 2000 to 2007, groundwater wells were again the primary source of water. In 2007, intermittent water was reestablished, and in 2011, improved infrastructure provided for better service of municipal water within area 1B (Waya, G., Northern Region Water Board (NRWB), personal communication).

### Questionnaire development and implementation^1^

A human factors questionnaire (available from the authors) included major sections addressing household use of water sources, sanitation sources, health information, water consumption patterns, and household socioeconomic information. Questionnaires were delivered orally in Chitumbuka and Chichewa, the common languages of the area,  and were accompanied by some observational measurements. In total, 51 households, representing 284 individuals aged 18–65 years, participated.

### Water quality monitoring

Microbial water quality parameters were determined for 30 shallow dug wells and linked to household drinking water. Questionnaires plus field observations were also recorded at each site. Samples of water stored in houses were collected directly from residents who participated in the questionnaire and considered representative of household drinking water conditions.

Microbial analyses were performed in duplicate within 8 h of water collection. New Petri plates, pipettes, and funnels were used for each sample, including for quality control samples. Forceps and the vacuum unit were wiped with alcohol and flamed between samples. Equipment contamination was checked by running blank water samples, using boiled water. Total coliform and *E. coli* were analyzed simultaneously using m-ColiBlue24^®^ (Hach Company, Loveland, Colorado). A 100-ml water sample was vacuum filtered through a 0.45-μm cellulose membrane. The membrane was transferred to a Petri plate containing a sterile absorbent pad saturated with 2 ml of broth and incubated at 35 ± 0.5 °C for 24 h. Water quality analysis was conducted at Mzuzu University Centre for Excellence in Water and Sanitation.

### Technology transfer

The purpose of the technology transfer phase of this project was to improve drinking water quality in two ways. First, project investigators trained local technicians and provided appropriate equipment to Mzuzu University. Second, the program provided water quality and hygiene information through local household presentations.

A major component of the technology transfer program was the training of eight technicians as questionnaire enumerators, water sample collectors, sample analysts, and household risk communication presenters. Although some technicians specialized, all were cross-trained. Infrastructure for analytical capacity to support water quality investigations is limited in developing countries due to a lack of reliable energy, unsterile conditions in existing laboratories, and difficulty in procurement of consumable laboratory supplies. For this investigation, analytical instruments with low to no municipal energy requirements were selected. For *E. coli* analysis, a field-style incubator was powered by a Goal Zero Sherpa 120 battery pack or car battery. Aseptic conditions were achieved for water sample analysis by covering a work table with plastic sheeting, rinsed daily with a 10 % bleach solution. Finally, analytical methods with low-cost consumables were selected. In developing countries with similar struggles as Malawi, Crane and Silliman [20] support a more frequent sampling strategy based on what may be considered lower quality instruments in areas where trained water quality technicians are not available.

The final step was the household risk communication program creation. A presentation was designed utilizing data collected from the household questionnaire, ground observations regarding the local conditions by the enumerators, and the water quality results. Household presentations took place at both households with safe water, based on WHO [21] guidelines, and at households with water posing a hazard to human health. Risk communication was targeted to the actual water users and decision-makers within the household. The delivery instructions and message used by the household risk presenters was guided by a script. The script included sections on general environmental public health information, sharing of groundwater and drinking water quality results of each household, discussion of how household-specific results may affect the health of infants and children, and discussion of simple actions to keep groundwater and drinking water safer at a household level. It also emphasized health benefits and cost savings from the use of clean water and low-cost, locally available, household treatment methods.

### Statistical analysis

All statistical tests were conducted using GraphPad Prism software version 5.04 for Windows (GraphPad Software, San Diego, CA, USA; www.graphpad.com).

## Results

### Human factors: results of questionnaire

In this section, we review some of the primary results from the household survey. The results showed the main source of household drinking water was municipal water delivered either by direct pipe to the dwelling, by piped water to the yard/plot, or from a public tap/standpipe. These municipal water supplies are serviced by the NRWB and are free of microbial contamination. Shallow well water was the main overall source of household water, but 75 % of respondents who use shallow well water reported that they did not drink the well water. Almost half of respondents had installed the shallow dug well under study due to piped water problems—that is, as a backup to the municipal water.

In this study, we inquired about possession of seven household accessories (bike, cell phone, radio, television, cook stove, refrigerator, and/or car) as an indication of household income/wealth. Respondents were grouped into two contingency categories (0–2 and 3–7 accessories) to represent lower and higher levels of household income, respectively. A Fisher’s exact test contingency table showed an equal likelihood that households in both income groupings were drinking municipal piped water or groundwater (*p* = 1.0000).

Eighteen percent of respondents reported that they were unemployed. A few households reported receiving income from people in the household working outside the area. No respondents indicated they were paid caretakers of animals using the shallow water supply studied. These observations are significant because they indicate the limited income available to purchase municipal water or household point-of-use treatment options and that shallow well water serviced household requirements rather than commercial interests.

Up to 20 houses were observed to be within 50 m of shallow dug well sites, showing the high density of area 1B. Most respondents stated that the well was used by many households; with only three of 51 respondents reporting that the shallow dug well was for a single household. Wells were observed to be as close as 1.6 m from the nearest house and 7 m to the nearest toilet. At many sites, there was more than one latrine within 30 m of the shallow dug well.

Regarding ownership and control of water sources, most respondents indicated the water was theirs, the property owners, or their neighbors. Only one respondent indicated the water belonged to the government. The respondent perception of ownership is noteworthy as legally all groundwater in Malawi is a public resource [18].

Based on the United Nation’s Millennium Development Goal, 94 % of households in the study area had an improved drinking water source. Here, improved drinking water sources included reported consumption of municipal water or groundwater from a semi-protected well. This incidence of using an improved drinking water source in area 1B matched the Mzuzu-wide 81.6 % population reportedly using improved drinking water [19].

Forty-five percent of respondents reported an adult woman is usually responsible to fetch the water for the household. This proportion is similar to the countrywide urban statistic that 51 % of adult women over the age of 15 are usually responsible for fetching the household water [22]. Most respondents reported gathering groundwater from the shallow dug well with a hard plastic container.

Most households reported shortages of drinking water, whether they consumed municipal water or groundwater (or both). Only 29 % of respondents reported having enough drinking water for the household every day of the year. The most commonly reported reasons for water shortages were long down times, irregular supply, low pressure, and failure to pay. Shortages were most frequently reported of several hours to several days during the months of September and October (dry season). Over half of households reported regularly storing water in their homes, either for daily use or to stockpile enough water for several days during times of shortages. The most commonly reported was a covered clay pot or plastic pail used to stockpile water within the household.

**Fig. 1:**
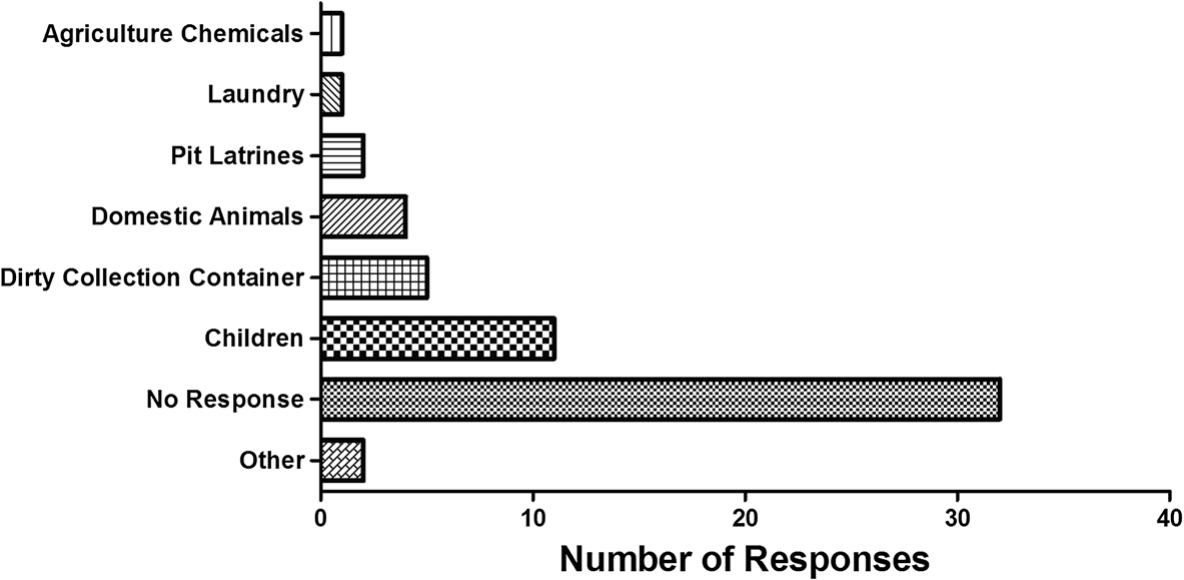
Household respondent reply to question “What do you think *(water source)* contamination is due mostly to?”

A key set of data linked risk and water quality when respondents were asked, “Do you think *(water source)* contamination is due mostly to ‘option X’”; they were offered six options and allowed to select as many categories as they wanted (Fig. 1). Only two respondents, each with at least a secondary education, identified the pit latrine near the well as a primary source of contamination. Yet, in the study area, latrines with an open pit and no slab were the most common toilet facility. Moreover, almost half of respondents shared their latrine with one or more households. Furthermore, project enumerators observed an open pit latrine was within 30 m of 27 of the subjects’ shallow dug wells.

The reported rate of occurrence of diarrhea was not associated with reported water source or treatment. Thirty-five percent of respondent households reported having a household child under the age of 5 with diarrhea in the 24 h preceding the field visit. Fisher’s exact test contingency table showed diarrhea was equally likely to be reported by households drinking municipal water or groundwater (*p* = 1.0000). In addition, Fisher’s exact test contingency table showed diarrhea was equally likely to be reported by households treating drinking water (either from municipal water or groundwater) by boiling or chlorine or not (*p* = 1.0000).

**Fig. 2:**
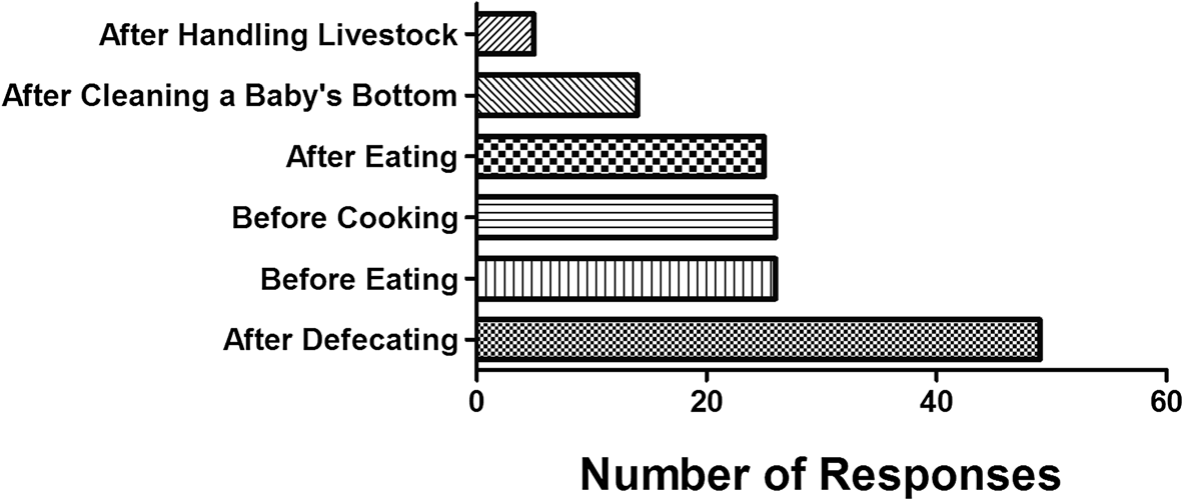
Household respondent reply to question, “When do you wash your hands with soap or other cleansing agent?”

Respondents were asked, “When do you wash your hands with soap or other cleansing agent?” They were offered six options and allowed to select as many categories as they wanted (Fig. 2). Most household respondents reported they washed hands with soap or other cleansing agents after toilet use. However, water for handwashing was directly observed by the research team to be available at only 4 of 30 households, and only 3 of 30 households had cleansing agents directly observed near the household toilet facility. Despite the high rate of reported handwashing, the lack of washing water and soap suggests handwashing after using the toilet is, in fact, uncommon. It seems likely the handwashing response is an instance of respondents answering what they determined to be the most acceptable answer rather than the most accurate answer.

### Microbiological water quality

Total coliforms were detected in groundwater from all 30 shallow dug wells (median of 1275 cfu/100 ml). Twenty wells were positive for the presence of *E. coli* (median of 14 cfu/100 ml). Total coliforms were also detected in 29 of 30 household drinking water samples (median of 52 cfu/100 ml), and *E. coli* was detected in 14 samples (median of 0 cfu/100 ml). Sixty-seven percent and 50 % of well water and household drinking water samples, respectively, exceeded the WHO [21] guideline of zero detections of *E. coli*.

**Fig. 3:**
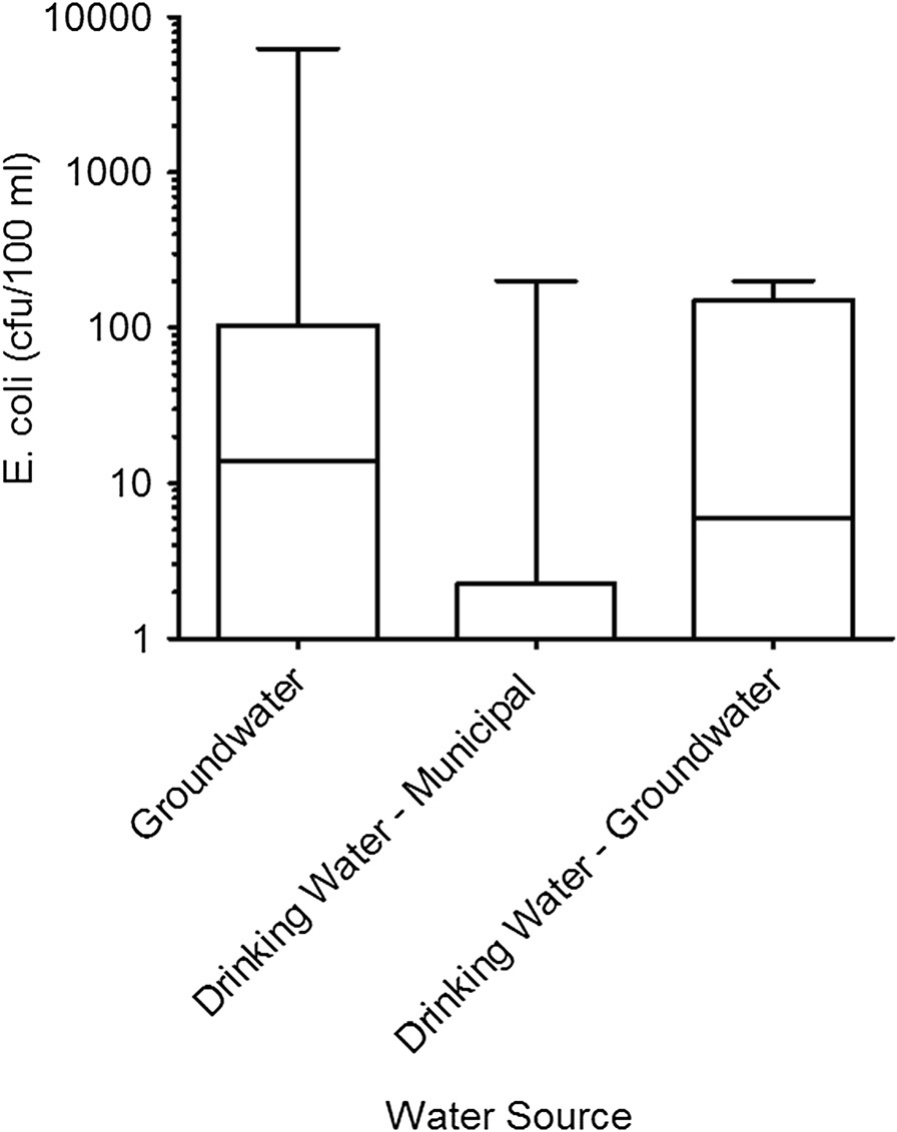
Distribution of *E. coli* counts in three sources of water. Box plots show 1–99th percentile data distributions

Median total coliforms and *E. coli* cfu were significantly different between groundwater and drinking water (Mann-Whitney test *p* values of <0.0001 and 0.014, respectively). Well water stored for drinking had significantly higher levels of *E. coli* than stored drinking water from municipal supplies (Fig. 3). The Kruskal-Wallis nonparametric ANOVA yielded an estimated *p* = 0.007. Dunn’s multiple comparison test showed median *E. coli* cfu in groundwater was significantly different than drinking water from a municipal source (*p* < 0.05).

Wells were 7 m to 30+ m to the nearest latrine, and 43 % were within 15 m or less. No wells complied with the Malawi Standards Board [23] for latrines to be at least 100 m from wellheads, though compliance is not necessarily practical in high-density settings.

Depth to groundwater was 0.5 to 10.9 m below ground surface. It was found 33 % of wells had water at 5 m below ground surface or less, though this may be groundwater and/or rainwater which leached through the thin soil layer. In the present study, linear regression analysis failed to show a significant correlation between *E. coli* detections and well depth (*p* = 0.181; regression coefficient of 0.06) or distance to a latrine (*p* = 0.330; and a regression coefficient of 0.035). Further statistical comparisons were made on contingency groupings of wells into below or above 5-m water depth and less than or greater than 15-m distance from a pit latrine. No significant differences in median *E. coli* cfu were detected. However, the maximum *E. coli* levels were associated with wells having the shortest distances to a latrine. In this study, latrine density and soil may be a more important factor than latrine spacing, though this needs further study.

*E. coli* tended to be higher in unprotected wells than semi-protected wells, but a Mann-Whitney test showed the difference in median cfu between the two groups was nonsignificant (*p* = 0.065). This may indicate moving up the drinking water ladder from an unprotected well to a semi-protected well is a good step but does not significantly improve water quality.

No shallow dug wells had a fence or enclosure. Few household small animals (dogs, chickens, pigeons) were present. Large animals (cows, pigs) were absent. This suggests animal waste is not the underlying source of *E. coli* environmental contamination.

### Household risk presentation

The nature of the household risk presentation included talking in the local language to individual households. Presentations were made orally within 1 month following administration of the household questionnaire and collection of water samples by the same group of technicians. Training sessions for presenters, including mock presentations, were conducted prior to household presentations. Households associated with the 30-well sites tested received a personal visit for purposes of risk communication, even if their water quality met the WHO guideline values for the constituents studied. Group presentations were not implemented as they were thought to inhibit questions or evoke embarrassment over potentially contaminated water quality results. The field technicians spent about 15–20 min per household. The presentation and time limits were designed to maintain participant interest. Field technicians had latitude to deviate from the script based on evolving needs.

Facilitators were sensitive to the ability of households to process the risk message. Some households desired more information, whereas others were overwhelmed by the same information. While the basic elements of the script were followed for all, if more information was sought, information was provided. At most households, a positive underlying message was used to educate about water quality health risks. However, some households required the message to be enhanced in that the consequences to human health of not protecting water resources were more dramatically communicated. Simple, nontechnical language was used in the presentations, assisted with pictorial aids. For example, *E. coli* cfu/100 ml was described as being the number of bacteria in 1/10th of a l000-ml container. The magnitude of the contamination problem was then conveyed in comparing the household result based on most adults drinking 2000 ml per day. By starting with an educational message, the water quality results were better received and the households better understood why they should care about water quality. At the conclusion of the presentations, a certificate of participation signed by the lead investigators was provided to each household participant, which included household groundwater and drinking water results.

A program evaluation 3 years after the technology transfer phase of this project has shown staying power and both the need and opportunity for water quality sampling. Over 1000 water samples have been sampled and analyzed by the originally trained technicians from water sources throughout Malawi since this project started in 2012.

## Discussion

Access to an improved water source does not equate to access to safe drinking water. There is a need for strategies that address greater access to safe water to be based on an understanding of human dimensions that inform decision-makers (including households) and help to initiate science-based behavioral changes among the local population.

The high diarrheal rate among children under the age of five years old in area 1B was not particularly associated with the source of drinking water and presence or absence of point-of-use treatment methods (boiling or chlorine). Groundwater quality degradation has been linked to well architecture and land use in sub-Saharan Africa [24], but was not shown in this study. In contrast, hand-washing practices were directly observed to be deficient and were inferred to be the source of drinking water bacterial contamination. Meta-analyses [4, 5, 25, 26] of the effect of handwashing on the occurrence of diarrheal diseases and studies monitoring contamination on hands alone suggest transfer between water sources and household storage or drinking vessels may be a major contamination route and thus explain why coliforms were ubiquitously detected in this study. One inference is handwashing should be encouraged in the study area to prevent the spread of waterborne disease, and higher in priority than point-of use water treatment.

Without the ability for regionally trained scientists to analyze water quality samples, how can an effective household risk communication program for safe water be developed? Technology transfer was an important risk communication component. In addition to direct communication about water quality with households, analytical laboratory equipment and technician training was provided to Mzuzu University that has been expanded in the 3 years since this project. Prior to this study, these field resources did not exist, showing the importance of linking both practice and theory in science education. Because risk communication depended on water quality data relevant to an individual’s own situation, it was necessary to develop analysis services nearby, wherever that may be. Three years after this study, the water quality laboratory is still aiding in providing this service. However, moving forward, it is acknowledged the laboratory will have a challenge in procuring water quality testing reagents. Limited foreign currency, high import duties, and lack of a foreign credit card mean Mzuzu University cannot straightforwardly procure supplies.

Research in other African countries has produced observations similar to those from Mzuzu. In Kenyan slums, Kimani-Murage and Ngindu [27], found total coliforms in 100 % of groundwater samples, and respondents identified the main source of contamination as children dipping dirty objects into the water source. Respondents’ perception of contamination caused by dirty objects overlooked the prevalence (38 %) of water sources located within 15 m of a pit latrine [27], similar to our study. The Kenyan study supports the idea that risk communication addressing respondents’ perception of contamination is essential to provision of safe water. The availability of improved drinking water sources has recently evolved in area 1B and may account for current perceptions of the source of waterborne contamination. Of particular note, professionals need water quality results to address risk communication. In developing risk communication programs, there should be regular plans for evaluating impact, which include household interviews tied to water quality assessments.

Risk communication entails important and complex relationships between environmental conditions, current risk perceptions, and economic impacts. Communicating the science of water risks in Malawi must be effective for long-term protection of human health and conservation of resources. The human dimensions of our risk communication program started with the design of the household questionnaire and the training of local university enumerator/technicians and continued with communication of water quality results back to the households. Taking into account the importance of human dimension information concerning the users and decision-makers in both household and quality of regional water supply, both are important.

## Conclusions

This paper informs the direction that the water sector needs to consider to provide safe drinking water. Additionally, area 1B is representative of other Malawian peri-urban neighborhoods. The technology transfer phase of this project aimed to improve drinking water quality through two objectives: Building infrastructure in the sciences to include water quality monitoring and increasing information sharing at a household level. Respondents had a perception of groundwater contamination. Yet, the most plausible cause of transfer of bacteria between the water source and consumption is likely due to a lack of handwashing and the resulting introduction of bacteria into the family’s water supply or periodically into the wells by children. This work suggests scale-up of risk communication programs follows RBCTT components in this study. Programs should include (1) obtaining sound technical measurements of all components of water use, from source to point-of-use, (2) integrating information about the human dimensions and water quality results in an overall risk communication program, and (3) construction of the risk communication program in partnership with established and knowledgeable regional health and development professionals, as well as (4) relevant technology transfer elements. Dr. Snow would have been pleased.

Although this study represents a limited regional study in Malawi, results indicate that, while improved water supply is available, safe drinking water is still not accessible to many households and this has a complex human and infrastructure dimension. Communicating the science of water quality and health risks in developing countries to meet the SDGs requires sample collection and analysis by knowledgeable personnel trained in the sciences, compiling baseline data, and, ultimately, an effective risk presentation back to households to motivate behavioral changes to effectively protect future water resources and human health.

## Footnotes

1 The Washington State University Institutional Review Board reviewed and approved the study for human subject participation.
